# Wealth, Unemployment, Social Investment, and Risk of ST-Segment Elevation Myocardial Infarction—An Ecological Analysis of a Low-Cardiovascular-Risk European Region

**DOI:** 10.3390/jcm14217707

**Published:** 2025-10-30

**Authors:** Elvira García-de-Santiago, María Lozano-Batuecas, Javier García-Pérez-Velasco, Jeny Gómez-Delgado, Daniel García-Arribas, Antonio Herruzo-León, Alberto García-Lledó

**Affiliations:** 1 Department of Biomedical Science, Universidad de Alcalá, 28801 Alcalá de Henares, Madrid, Spain; elvgarcia2@telefonica.net; 2 Department of Medicine and Medical Specialties, Universidad de Alcalá, 28801 Alcalá de Henares, Madrid, Spain; 3 Department of Cardiology, Hospital Universitario Príncipe de Asturias, 28805 Alcalá de Henares, Madrid, Spain

**Keywords:** acute myocardial infarction, wealth, unemployment, public investment

## Abstract

**Objectives**: A retrospective ecological study was conducted to analyze the relationship between the incidence of myocardial infarction with ST-segment elevation (STEMI) and various sociodemographic factors in municipalities within the Community of Madrid, a high-income and low-cardiovascular-risk European region. **Methods**: This study analyzed a database of patients registered in the regional network for STEMI care from January 2014 to December 2018. Thirty-four municipalities with populations greater than 10,000 inhabitants were included. The mean annual incidence of STEMI (iSTEMI) was estimated for each locality, and several variables of wealth, employment and social investment were obtained from public databases. **Results**: During the period of the study, 2561 confirmed STEMI cases were recorded in the selected localities, with an average incidence of 23 events per 100,000 inhabitants and year. The mean age was 62, with 83% of patients being male. Among municipalities included in the study, a significant direct correlation was found between iSTEMI and unemployment rate (r = 0.354, *p* = 0.04). A significant inverse correlation was found with all wealth-related variables, mainly with a composed deprivation (poverty) index (r = −0.624, *p* < 0.001) and the percentage of employees in the financial sector (r = −0.497, *p* = 0.003). No correlation was found between iSTEMI and the sociodemographic or public investment variables retrieved. Multiple regression analysis showed that the model best fitted when energy billed per inhabitant and mean income tax per taxpayer were introduced. **Conclusions**: Residents of areas with lower incomes and higher unemployment rates may be at a greater risk of STEMI. This should be taken into account when planning cardiovascular prevention and community health management.

## 1. Introduction

Cardiovascular disease, specifically ischemic heart disease (IHD), is one of the leading causes of illness and death worldwide [1]. There is ample evidence linking cardiovascular disease to lower income at national and regional levels [2,3]. At an individual level, the European Society Guidelines on cardiovascular disease prevention recognize low income as an independent risk factor [4], as demonstrated by studies which also consider the educational level to be a factor associated with a higher number of events [5]. However, the increased risk cannot be attributed exclusively to income or education, as they may depend on complex interactions with other individual factors associated with wealth, such as diet, physical activity and stress, as well as population factors, such as pollution levels and public investment in social and health resources in subject environment.

ST-segment elevation myocardial infarction [STEMI] is a major cause of morbidity and mortality among cardiovascular diseases, most frequently affecting individuals of working age [6,7]. This is a vital period when employment type is relevant, and when individual income is usually higher than in older age groups, where other forms of IHD are more common [7]. Conversely, unemployment during working age can lead to stress, reduced income and depression, which can increase cardiovascular risk [8]. While most published studies focus on the relationship between income and cardiovascular disease as a whole, other studies analyze the relationship with IHD rather than its specific manifestations. Therefore, it would be interesting to study the potential relationship between indicators of income, employment and social investment in relation to STEMI, a specific form of IHD. This condition has its own pathophysiological and epidemiological features, as well as a specific and costly clinical management [9,10].

From an ecological perspective, this study aims to analyze the relationships between income level, employment, markers of public investment in education and health, and the incidence of STEMI in various municipalities in a high-income and low-cardiovascular-risk European region.

## 2. Patients and Methods

### 2.1. Population and Study Design

A retrospective ecological study was conducted to analyze the relationship between the incidence of STEMI and various sociodemographic factors in populations of more than 10,000 inhabitants in the Community of Madrid (CM), Spain, excluding the capital. Given the epidemiology of STEMI, only people over the age of 18 were included in the reference population. The CM is a small region (8030 km^2^) with around 7 million inhabitants, most of them evenly distributed between the capital, Madrid, and the towns in the metropolitan area. In 2022, it ranked in the top 80% of European Union regions in terms of gross domestic product (GDP) per capita in purchasing power parity [11].

From a healthcare perspective, it has a universal, free public system comprising a network of 42 hospitals, 266 health centers and 163 local medical clinics [12]. In 2018, the standardized cardiovascular mortality rate in the CM was the lowest in Spain at 168.8 per 100,000 inhabitants, placing it among the lowest rates in developed countries [13].

To avoid random effects due to the size of smaller populations, 34 municipalities with more than 10,000 inhabitants were selected. Madrid, the capital city, was also excluded from the study because of significant income disparities between its districts, since the available database did not permit distinctions to be made between them.

### 2.2. Sample Analyzed

This study analyzed a sample of patients with STEMI recorded in the “Código Infarto Madrid” (CIM) registry between January 2014 and December 2018. CIM is a healthcare network that organizes the management of STEMI, primarily through primary angioplasty, in the Madrid region and it is described in detail elsewhere [6]. Data on CIM patients are collected in an official registry which must include basic demographic information, the date and time of symptom onset and the date, time and location of final treatment. This is subsequently supplemented with information on coronary angiography and angioplasty, if performed, and survival during hospitalization. The registry does not include data on risk factors, previous cardiac history, treatment or post-discharge outcomes. Activation of this system does not necessarily imply that the patient suffers from STEMI. For this reason, only confirmed cases of STEMI in which a culprit plaque had been identified by angiography were analyzed. Therefore, all of them were type I myocardial infarctions according to the fourth universal definition of myocardial infarction [14].

The database was independently audited between 2014 and 2018, which is why this period was selected for the study. The study was approved by the ethics committee of one of the reference centers of the Madrid STEMI Code network (Príncipe de Asturias University Hospital) as an extension of a previous code OE 10/2017, dated 20 December 2023.

### 2.3. Data Sources

After obtaining authorization from the ethics committee of one of the hospitals participating in the CIM network, an anonymized version of the CIM registry was obtained from the authority that maintains the database. This version contained information on the municipality of residence, age, sex, the date and time of symptom onset, the date and time of healthcare attendance, type of care provided and findings. Access to patients’ addresses or the location where the episode occurred was not granted by the authority in charge of the original database, as this was deemed to pose a privacy risk.

The primary source used to obtain sociodemographic data on municipalities was the public database compiled by the Statistical Institute of the Community of Madrid [15]. The following variables were collected between 2014 and 2018:

- Sociodemographic variables: population, mean age, rate of ageing, female-to-male ratio, average income, gross domestic product per capita, unemployment rate, percentage of people employed in financial services, number of foreign immigrants per 1000 inhabitants.
- - Social investment: public hospitals in the population, non-university students per teacher/school unit, health centers per 10,000 inhabitants, public libraries per 10,000 inhabitants.

In order to obtain a single indicator of deprivation (poverty) previously validated in the Spanish population, the deprivation index defined by the Spanish Society of Epidemiology was obtained for each population. This index considers six variables in a weighted manner: manual and temporary workers, unemployment, insufficient education overall and in young people (aged 16 to 29 years), and dwellings without access to the internet [16,17].

### 2.4. Statistical Analysis

The incidence of STEMI in each municipality was calculated based on the number of cases recorded among the population over 18 years of age each year.

The Kolmogorov–Smirnov test was used to analyze the distribution of the variables. For normally distributed variables, the data are summarized as mean and standard deviation. For non-normal variables, the median and range are used.

A longitudinal panel was created for fixed effects linear regression analysis. The enter method was chosen. The Durbin–Watson test was used to identify potential autocorrelation problems, and Savin and White tables were used to limit the number of appropriate variables to ensure the validity of the models explored.

Two different models were created. First, all the independent variables retrieved from the public database [15] shown in Table 1 and Table 2 (please see results) were used. The second model used the deprivation index instead of the different wealth-related variables with all the other independent sociodemographic and public investment variables.

For correlation studies and graphics, the data were collapsed using the average annual value for each variable and municipality. A database was constructed in which the average annual STEMI incidence and sociodemographic values were assigned to each municipality. Correlation between continuous variables was analyzed by the Pearson correlation coefficient.

To analyze the potential effect of the only discrete variable included in the study (presence or absence of a public hospital) on iSTEMI, Student’s *t*-test for unrelated samples was used.

To avoid leverage due to the different sizes of municipalities, a sensitivity analysis was conducted in three steps: First, by excluding those with extreme values. Second, Cook’s distance was calculated for each variable that was significantly correlated with the dependent variable, and extreme values of Cook distance were removed. Finally, we obtained the correlation among independent variables and the logarithm of the dependent one.

IBM SPSS Statistics 29 (Armonk, NY, USA: IBM Corp.) was used for statistical analysis. Mapitude 2025 software (Newton, MA, USA: Caliper Corp.) was used to analyze spatial autocorrelation using Moran’s I test and to create distribution maps of the variables.

## 3. Results

Based on data from 2018, the reference population for the study consisted of 2,250,632 people over the age of 18 residing in 34 municipalities with more than 10,000 inhabitants in the Autonomous Community of Madrid, 52% of whom were male.

Between 2014 and 2018, the CIM registry recorded 2561 confirmed STEMIs with culprit plaque in the selected municipalities. This equates to an average incidence rate of 22.98 events per 100,000 people per year, ranging from 14 to 45 cases per 100,000 people across the different localities. Of these events, 83% occurred in male patients with a median age of 62 years (rank 24 to 96).

Table 1 and Table 2 summarize the centralization and dispersion values for sociodemographic variables, income, employment and public investment in the different municipalities. Despite the region’s small size, differences between municipalities are evident, particularly in terms of wealth and employment type, and less so in terms of average population, age and teacher-to-student ratio. Regarding iSTEMI, the maximum incidence rate (44.96 per 100,000 inhabitants) was three times higher than the minimum recorded.

**Figure 1:**
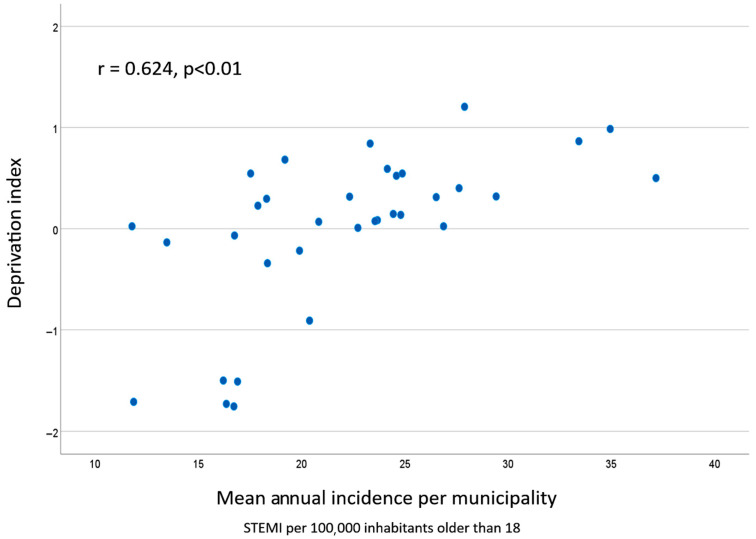
Scatterplot graphic showing the correlation between the deprivation index and the average annual incidence of STEMI in the 34 municipalities included in the study.

**Figure 2:**
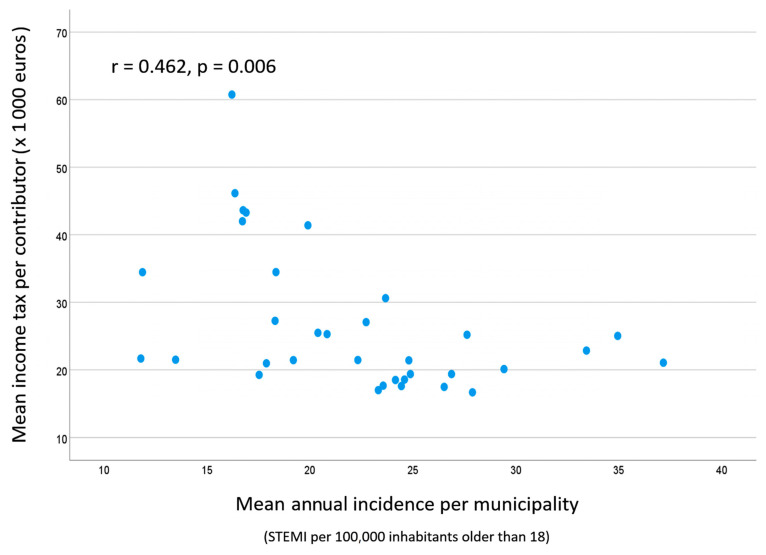
Scatterplot graphic showing the correlation between mean income tax per contributor and the average annual incidence of STEMI in the 34 municipalities included in the study.

Table 3 shows the correlation between iSTEMI and the annual mean values of the variables included in the study. No correlation was found between iSTEMI and the sociodemographic or public investment variables except for mean age (r = 0.426, *p* = 0.012). Conversely, a significant direct correlation was found with the deprivation index selected (r = 0.624, *p* < 0.01; see Figure 1), and an inverse correlation with all wealth variables and the number of employees in the financial sector. Figure 2 shows the correlation between iSTEMI and the average income per taxpayer.

The sensitivity analysis ruled out a significant confounding effect caused by the different sizes of the municipalities included in the study. When those with more than 150,000 inhabitants were excluded (six towns), the correlation between the deprivation index and mean incidence was stable (r = 0.646, *p* < 0.000 before and r = 0.652 after). The same was observed when calculating Cook’s distance or analyzing the correlations between the independent variables and the logarithm of the dependent one.

**Figure 3:**
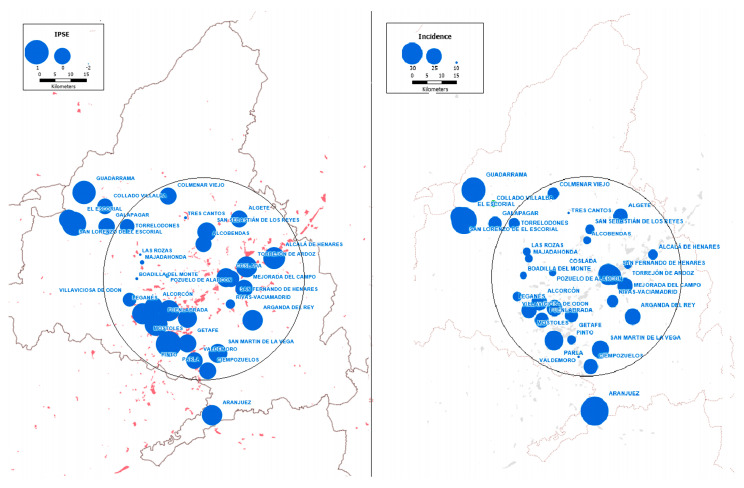
Maps show the spatial distribution of data relating to populations of over 10,000 inhabitants in the Community of Madrid. The circle indicates a 35-km radius around the capital city, which was excluded from the study. Red shaded areas indicate industrial facilities. The size of the blue dots correlates with the magnitude of the variables. Deprivation index (IPSE) data is shown on the left and incidence of STEMI in people over 18 years of age (incidence) is shown on the right.

A map was created with the deprivation index data and incidence of STEMI to explore the spatial distribution of both variables (Figure 3). A circle with a radius of 35 kilometers has been drawn to illustrate the spatial distribution of municipalities around the capital. The distribution of industrial areas is shaded in red. There is a spatial correlation between populations with the highest deprivation index (IPSE) and those with the highest incidence of STEMI. Notably, populations in the northwestern region near the capital have a very low deprivation index and incidence of STEMI, whereas populations further away have higher indices and incidence rates. Both variables coincide spatially with industrial facilities, which are concentrated in the southern and south-eastern areas of the Community of Madrid. When Moran’s I autocorrelation test was performed, a significant value close to 1 was found for most wealth variables, as well as for incidence of STEMI, while no significant values were found for public investment variables

Linear regression analysis applied to the longitudinal panel with the yearly data showed that the deprivation index was the only independent variable in the model significantly associated with the incidence of STEMI (*p* = 0.022). When the other variables of wealth or poverty were included, the only significant independent variable in the model was GDP per capita (*p* = 0.040).

Of the 34 municipalities, 16 had a public hospital, and 1 had two. No difference was observed in iSTEMI according to the presence or absence of a hospital in the municipality.

## 4. Discussion

Our study shows that, even in a small territory and in a population with low cardiovascular risk, markers associated with employment and wealth appear to be associated with the risk of suffering a specific cardiovascular event, such as STEMI. In contrast, most demographic variables or those derived from public investment in health or education were not associated with this risk. 

From an ecological point of view, a close association is observed worldwide between the wealth of different countries and territories and the risk of disease and death, including those of cardiovascular origin [3]. While the incidence and mortality of cardiovascular disease have declined in recent years in high- and middle-income countries, they continue to increase in low-income countries [18]. The differences observed between high- and low-income countries affect not only the incidence of events, but also the treatment received and survival after suffering them [19]. Our study is consistent with the existing literature and shows that, at the ecological level, these differences in risk can be observed even within a small territory with a universal healthcare system and high overall income. The increased risk in different municipalities was mainly associated with the type of work, unemployment rates, and average tax return value of residents. These findings align with the literature and support the idea that low socioeconomic status is an individual risk factor. Furthermore, our data do not suggest a relationship between the social investment indicators included in the study and the risk of STEMI. In other words, deprivation appears to be a risk factor that persists despite public investment in health or education systems.

Analyzing the different variables in terms of both geographical distribution and normality reveals completely different patterns for wealth and deprivation variables and for demographic and public investment ones. The latter follow a normal statistical distribution in almost all cases and are distributed evenly across the territory. In contrast, both wealth variables and the incidence of STEMI are non-normally distributed and appear concentrated mainly in two areas (Figure 3): those furthest from the capital city and those in areas of greater industrial concentration in the south and south-east of the region. This distribution suggests that public investment is similar across all municipalities but does not increase in areas of greater need. It also suggests other possible associations, such as housing prices (which are usually cheaper the further away from the metropolis) or environmental pollution, which are discussed below.

The European guidelines for cardiovascular prevention [4] recognize low socioeconomic status as an independent risk factor for atherosclerotic cardiovascular disease, although they do not consider this population as a specific intervention group. However, this subpopulation may have less control over risk factors [20] and lower adherence to cardiac rehabilitation programs [21]. Also, this population may be more vulnerable because they are more likely to suffer from work-related stress and depression due to unemployment or low wages [22]. Our results support the relationship between the risk of STEMI and both unemployment and the type of work performed. The reduced availability of resources makes it more difficult to afford chronic primary and secondary prevention treatments [23]. Following primary angioplasty, patients require prolonged antiplatelet therapy involving expensive drugs that require strict adherence [24]. Therefore, low educational levels and lack of income may limit the benefits of STEMI reperfusion strategies, although the data from this study cannot support this conclusion. A randomized study involving low-income individuals over the age of 65, conducted in the province of Alberta (Canada), showed that the removal of pharmaceutical copayments for major cardiovascular drugs increased adherence but did not reduce events [25]. The characteristics of the population over the age of 65, for whom copayments are usually lower and often limited, may have affected the study results. These results could have had a greater impact on a younger working population whose copayments are usually higher, as is the typical patient with STEMI. The relationship between lower incomes and lower educational levels and poorer health habits should also be considered, as these factors would limit the isolated effect of reducing healthcare costs.

Studies have shown that people with lower levels of education are at an increased risk for cardiovascular disease [5,26] in both the United States and Europe [5]. Educational attainment can affect health in many different ways, including through income levels, better access to healthcare systems when they are not universally covered, and improved health education [5]. Our study shows that differences in the incidence of STEMI do not appear to be related to markers of public investment in educational resources, whether considering the number of teachers per student or libraries per inhabitant.

Air pollution is an environmental factor associated with an increased risk of myocardial infarction, primarily due to an increase in suspended microparticles [27,28]. Due to its relationship with various diseases, air quality can be a confounding factor in studies that consider poverty rates and disease risk. However, it can also be considered another consequence of inequality [29]. Pollution variables have not been included in this study because data are only available for 16 of the 34 analyzed municipalities, and the wealth and public investment data are only available annually, which is an inadequate time interval for analyzing the effect of pollutants on health. Nevertheless, the spatial association evident in Figure 3 between industrial areas (shown in red) and the incidence of STEMI does not contradict the existing literature and could prompt further research in this area.

### 4.1. Limitations

As this is a retrospective ecological study, its value is limited to the possibility of generating hypotheses. The results cannot be interpreted at an individual level, given that the homogeneity of the populations analyzed should not be assumed [30]. The CIM database does not collect sociodemographic data on each patient, so it has not been possible to analyze personal data. For confidentiality reasons, we were not permitted to know the exact place of residence of the patients. Assigning cases by district, especially in the city of Madrid, would have enabled us to include more cases.

The number of events per year was very low in smaller municipalities, with the biggest municipality being 13 times larger than the smallest. To avoid errors due to leverage and heteroskedasticity, a sensitivity analysis was performed with consistent results.

Wealth variables are often closely related to each other. The existence of autocorrelation can limit the significance of the results. To reduce the confusion caused by multicollinearity and autocorrelation, the number of variables was limited after assessing their effects using the Durbin and Watson test, and a single deprivation index validated by the Spanish Society of Epidemiology was used. In both models, the results were consistent, showing a relationship between the risk of heart attack and at least one of the wealth/deprivation parameters, but not with those of public investment.

Spatial autocorrelation may limit the significance of the results. However, the fact that no significant value was found in the Moran I test for any of the public investment variables leads us to believe that public investment does not follow a pattern linked to other variables (such as the health status of a municipality or its poverty rates).

The CIM database does not collect data on STEMIs managed in private hospitals in the CAM. The treatment of higher-income individuals in these hospitals could create a false impression of an increase in the incidence of STEMI among lower-income individuals. However, Spain’s healthcare system is universal, and emergency cases are attended by public out-of-hospital emergency services, which transfer patients to public hospitals if a STEMI is suspected. According to protocol, these services refer patients to hospitals in the CIM network. Private healthcare does not have these out-of-hospital services, so STEMI cases treated in private centers are limited to patients who arrive on their own, and only those who have a primary angioplasty alert.

### 4.2. Strengths

Uniquely, the study includes a single type of cardiovascular event, STEMI, in which plaque rupture has been demonstrated, and therefore is undoubtedly a type I infarction. Our results demonstrate the relationship between a single pathophysiological phenomenon (the vulnerability of coronary atheromatous plaque) and the analyzed variables. The database used has been audited, so the incidence of STEMI could have been underestimated, but not overestimated. Therefore, the statistical relationships described are less likely to be due to chance.

## 5. Conclusions

In a small region with high average income and low cardiovascular risk, the incidence of STEMI is higher in municipalities where the population’s average income is lower, the unemployment rate is higher, and the proportion of employees in the financial sector is lower. Conversely, there does not appear to be a relationship between indicators of public investment in health or education and the incidence of STEMI. This should be taken into account when planning cardiovascular prevention and community health management.

## Figures and Tables

**Table 1:** Distribution of the variables analyzed across the sample of municipalities included in the study. Normally distributed variables.

	Mean	Minimum	Maximum	Standard Deviation
**Sociodemographic data**				
Mean age	39.1	35.2	42.5	2.01
Rate of inhabitants older than 65	13.6	6.2	20.9	3.51
Rate of female population (%)	37.2%	33.8%	41.2%	1.74
**Public investment parameters**				
Rate of teachers per non-university alumni	13.11	11.57	14.39	0.74
Public health centers (per 10,000 inhabitants)	1.13	0	4.94	1.16

Data represents the central value and the value of each parameter in the municipality with the lowest and highest data.

**Table 2:** Distribution of the variables analyzed across the sample of municipalities included in the study. Non-normally distributed variables.

	Median	Minimum	Maximum	Rank
Incidence of STEMI (per 100.000 inhabitants)	22.5	14.11	44.96	30.85
**Sociodemographic data**				
Inhabitants aged 18 or older	60,251	12,763	170,160	150,397
Foreign population (per 1000 inhabitants)	117.78	69.1	235.2	184.1
**Wealth and employment data**				
Deprivation index	0.20	−1.98	0.62	2.60
Mean income tax per taxpayer (×1000 euros)	21.6	16.8	62.7	51.4
Gross domestic product per capita (×1000 euros)	22.2	10.6	68.2	57.6
Unemployment rate (%)	7.7	3.45	10.40	6.95
Financial employees (per 1000 inhabitants)	16.3	9.3	52.6	42.9
**Public investment parameters**				
Libraries per 10,000 inhabitants	0.37	0.14	0.87	0.73

Data represents the central value and the value of each parameter in the municipality with the lowest and highest data.

**Table 3:** Correlation among the mean incidence of myocardial infarction with ST-segment elevation (miSTEMI) and demographic, wealth, employment and public investment variables in the 34 municipalities included.

Demographic Data					
	Population older than 18	Mean age	Older than 65	Female	Foreign population
r	−0.114	0.426	0.339	0.114	0.272
*p* value	ns	0.012	ns	ns	ns
**Wealth and Employment Data**					
	**Income** **taxes**	**GDP**	**Unemployment** **rate**	**Financial** **employment**	**Deprivation index**
r	−0.462	−0.442	0.354	−0.497	−0.624
*p* value	0.006 *	0.009 *	0.040 *	0.003 *	<0.001 *
**Public Investment Data**					
	**Rate of alumni per teacher**		**Libraries per 10.000** **inhabitants**		**Health centers per 10.000 inhabitants**
r	−0.052		−0.204		0.308
*p* value	0.771		0.248		0.076

*: significant correlation; GDP: gross domestic product per inhabitant (per municipality); iSTEMI: mean incidence of STEMI (per municipality). Ns: non-significant.

## Data Availability

Dataset available on request from the authors.

## Abbreviations

The following abbreviations are used in this manuscript:

CM	Community of Madrid
CIM	Código Infarto Madrid
GDP	Gross domestic product per capita
IHD	Ischemic heart disease
iSTEMI	Average incidence rate of STEMI
STEMI	ST-segment elevation myocardial infarction
